# Efficiency of Hysteresis Rods in Small Spacecraft Attitude Stabilization

**DOI:** 10.1155/2013/459573

**Published:** 2013-12-31

**Authors:** Assal Farrahi, Ángel Sanz-Andrés

**Affiliations:** Instituto Universitario de Microgravedad “Ignacio Da Riva” (IDR), ETSI Aeronauticos, Universidad Politécnica de Madrid, Plaza del Cardenal Cisneros 3, 28040 Madrid, Spain

## Abstract

A semiempirical method for predicting the damping efficiency of hysteresis rods on-board small satellites is presented. It is based on the evaluation of dissipating energy variation of different ferromagnetic materials for two different rod shapes: thin film and circular cross-section rods, as a function of their elongation. Based on this formulation, an optimum design considering the size of hysteresis rods, their cross section shape, and layout has been proposed. Finally, the formulation developed was applied to the case of four existing small satellites, whose corresponding in-flight data are published. A good agreement between the estimated rotational speed decay time and the in-flight data has been observed.

## 1. Introduction

Although the technology of Passive Magnetic Attitude Stabilization Systems (PMASS) started to be developed in 1961 [1, 2] and has been applied to several small size satellites [3], there are still some issues to be solved regarding this system, such as the sizing of its system parameters and predicting the in-orbit performance without going through long experimental procedures. These problems are rooted in the difficulties that exist in determining the magnetic characteristics of the ferromagnetic bodies (hysteresis rods) that are applied as damping devices on-board the satellites, as the variation of these characteristics is a complex function of different variables that might not be very well known.

One of the earliest investigations in this field was carried out by Fischell [4]. According to this work, the magnetic properties of the ferromagnetic bodies, apart from the properties of the material, were seen to be very sensitive to the body length-to-thickness ratio (elongation). Through experimental results of Fischell [4] it was seen that the rod with higher value of elongation presented a larger damping capacity.

Also the cross-section shape and size of the rods are the other known factors that strongly affect the magnetic properties of the hysteresis rods, as the damping capacity of a hysteresis rod is directly proportional to its volume. Therefore, for rods with the same shape and elongation, the bigger the rod is, the larger its corresponding damping capacity will be. Evidently, the length of the rod cannot exceed the lateral dimensions of the satellite. Thus, for increasing the volume of the body its other dimensions should be increased. However, increasing the thickness leads to a reduction of elongation and consequently damping capacity of the rod. Considering these effects together, it is expected that an optimum value for the thickness of the hysteresis rods exists, for which the corresponding efficiency of the rod is a maximum. This problem has been recently studied to some extent in [5], in which the energy dissipated by Thin Film Rods (TFR) made up of different materials has been studied and the corresponding optimum thickness has been evaluated for different materials. By TFRs we are referring to rods with rectangular cross-sections and a thickness less than 1 mm.

In addition to the material, shape, elongation, and volume, experimental data show that the heat treatment and manufacturing process are also parameters that influence the magnetic properties of ferromagnetic bodies; however, the exact influence cannot be predicted theoretically yet [6, 7]. Some experimental results concerning the variation of the magnetic properties of certain materials under the influence of some manufacturing and heat treatment procedures are presented and analyzed to a certain degree in [6]. Also, some interesting results regarding manufacturing process were obtained during the design phase of EduSAT [7], which showed that it is possible to obtain almost the same magnetic properties for rods that were manufactured from the same material but through two different manufacturing processes.

Another important parameter that affects the efficiency of the on-board hysteresis rods is their corresponding on-board layout arrangement with regard to the other magnetic materials in place, specially the permanent magnet. In-flight data of the satellite Delfi-C3 shows the importance of the special care to be applied during the design of the on-board layout arrangement of the rods with respect to other magnetic materials on-board the satellite. During the design, it was predicted for the Delfi-C3 cubesat that its initial angular velocity will be reduced to the desired one within a few hours, while in flight it took the satellite about 3 months [8]. This problem has been analyzed in the current paper and has been found to be mainly due to the relative position of the rods with regard to the permanent magnet, which led the rods to get saturated and not being able to perform as predicted.

It is also known that parallel rods have some mutual influence on each other, effect that should be taken into account. This problem was also studied to a certain degree in [5]. It is also known that in the arrangement of several permeable rods along the same direction, there exists an optimal elongation that reduces this mutual influence to a minimum, as it was implemented in the design procedure of the PMASS of EduSAT [7], MUNIN [9], TNS-0 [10], and Reflector [11].

In this paper, the magnetization process inside the ferromagnetic bodies is analytically modeled. Based on this model, the best cross-section shape, elongation, and position, regarding efficiency, for the hysteresis rods in satellites with different sizes is proposed. Further, a simple analytic model for evaluating the corresponding damping time is proposed. The theoretical results are compared with the available in-flight data of different satellites, showing a good agreement.

This paper is organized as follows: in Section 2, the domain theory in ferromagnetic materials, which is the base where the hysteresis phenomena in soft ferromagnetic materials can be justified, is summarized. In Section 3, the mathematical models are presented for the following terms: (a) the initial magnetization curves of soft ferromagnetic materials, (b) the magnetic field generated by a permanent magnet, (c) the hysteresis losses of a ferromagnetic rod with a defined shape, (d) the demagnetizing field of a hysteresis rod with a defined shape, and (e) the rotation speed decay time of a satellite.

In Section 4, the results obtained based on the proposed mathematical models are presented and the following points are discussed: (1) the optimum on-board layout arrangement for different shapes of hysteresis rods with respect to the permanent magnet, (2) an estimation for the initial magnetization curve of different ferromagnetic materials, (3) the variation of the hysteresis loss versus some of shape characteristics of different ferromagnetic materials, (4) the evaluation of the optimum thickness and diameter for both TFR and Circular Cross-Section Rods (CCSR) for a given length of the body, and (5) the evaluation of rotational speed decay time of four existing satellites and the comparison with their respective in-flight data to assess the proposed model. In the last point, the original designs are compared with the optimum designs obtained based on the optimum hysteresis rod shape and size for different sizes of satellites, in order to study the efficiency of the original designs.

And finally, in Section 5 conclusions are drawn.

## 2. Hysteresis and Domain Theory

In this subsection, the application of soft ferromagnetic bodies to the PMASS is explained and the domain theory of ferromagnetic materials, which is the base where the hysteresis phenomena in soft ferromagnetic materials can be explained, is summarized.

While an Earth orbiting satellite is in its initial rotation mode, the hysteresis rods mounted on it experience a time varying magnetic field. The interaction of the on-boarded hysteresis rods with the time varying magnetic field causes some part of the angular kinetic energy of the satellite to be transformed into heat that is produced inside the hysteresis rod, that is produced inside the hysteresis rod. This transformation of energy leads to a decay of the initial rotational speed of the satellite. The generated heat is due to the irreversibility of the magnetization process in soft ferromagnetic materials. This irreversibility causes the variation of the magnetic flux density generated inside the hysteresis rod, *B*, due to a time varying applied field, *H*, to follow a hysteresis loop, and consequently produces hysteresis losses. It is known that the amount of hysteresis losses is proportional to the enclosed area within the loop. Therefore, in order to estimate the rotational-speed decay time of the satellite, an estimation of the enclosed area within the hysteresis loop of the rod is required. For doing so, the several steps of the magnetic domain process that the material goes through have to be known. Depending on the domain process type the respective losses are found to be a different function of the applied magnetic field. The changes involved in the domain process can be explained with the help of the initial magnetization curve of the materials. In order to explain the different domain process types using the magnetization curve, the basics of domain theory should be known; therefore, a short summary is included in the following paragraphs for reference.

As it is known [6], the domain theory in its simplest form states that every ferromagnetic material is composed of many regions that are magnetized up to saturation but aligned in different directions. In the demagnetized state, these regions are distributed at random directions, which leads to a zero net magnetization of the specimen. These regions are called magnetic domains, and its evolution is defined as domain process in this paper, which is explained in the following paragraphs.

The existence of magnetic domains can be explained by the tendency of the material to stay in its lowest possible level of energy. In the demagnetized state or the magnetized states below the saturated state, the decrease in the magnetostatic energy composing magnetic domains is greater than the energy to form magnetic domain walls, so multidomain specimens arise. By applying an external magnetic field to a demagnetized specimen, the direction of the magnetization vector of the domains starts to change, following a certain pattern.

**Figure 1:**
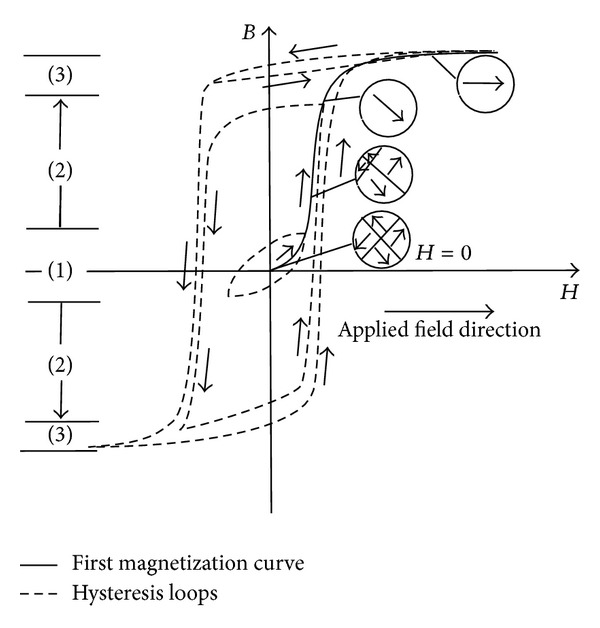
Variation of the magnetic flux density, *B*, as a function of the external applied magnetic field, *H*, with different peak values. Depending on the applied field the variation follows a different loop, which divides the magnetization process into 3 regions: (1) reversible domain boundary displacement, (2) irreversible domain boundary displacement, and (3) rotational motion of the domains. Inserts: domain configuration during several stages of magnetization along the first magnetization curve [6, 17].

According to the domain pattern of change, the initial magnetization curve of the materials is divided into three main parts as is shown in Figure 1. By studying the microstructure of the ferromagnetic bodies, it was observed that by increasing the external magnetic field applied to a body from zero to its maximum value, first the walls of the domains start to displace. This displacement happens in such a manner that the volume of the domains that have the magnetic direction orientation in the favor of external magnetic field increases, and the volume of domains with the magnetic direction oriented in the opposite direction of the applied field shrinks (region 1).

This displacement of the wall is reversible at first, but by increasing the strength of the applied field, the displacements enter an irreversible phase. Furthermore, by increasing the amplitude of the external magnetic field, the magnetization vectors of the domains start rotating to get aligned with the easy magnetization axis closest to the external field (region 2). Increasing the strength of the external magnetic field even more, the magnetic moments that were already aligned with the preferred easy magnetization axis gradually rotate into the field direction, leading the material to a single domain sample of ferromagnetic specimen (region 3).

This motion of domains is accompanied with friction which is the result of imperfections in the form of impurities in the composing elements or dislocations in the material. A part of the energy absorbed by friction is spread along the material in the form of heat. This energy loss leads to the hysteresis phenomenon in ferromagnetic materials. The first part of the initial magnetization curve, which has an upward concavity and corresponds to the displacements of domain boundaries, was first modeled by Rayleigh relationship [6]. The corresponding hysteresis losses of this part are about 0.004 times the maximum hysteresis losses that can be dissipated in the material. Therefore, during the design procedure of a PMASS, it is preferable to define the baseline operation point away from this part of the curve. Since the goal is to design a system with the highest possible energy dissipation capacities. The second part of the magnetization curve, which is referred to as the irreversible displacement of the domains' walls, has the maximum permeability, *μ*. The maximum losses of the material happen when the point which represents the magnetization variation of the material in *B* − *H* diagram traces the hysteresis loop in this portion of the curve. The whole domain process, along with the initial magnetization curve and the corresponding hysteresis loops that occur as a result of time varying magnetic field, is schematically illustrated in Figure 1.

This hysteresis effect has been used as a simple and reliable means for damping the spinning and oscillating motions of an Earth orbiting satellite. However, as the damping characteristic of the hysteresis rods is a complex function of several parameters, its estimation is not a trivial task. As is mentioned in the introduction, apart from the material, the size and shape of the rod, its manufacturing process, and also its corresponding layout with respect to other magnetic components on-board the satellite have an influence on the damping efficiency of the rods. In the next section the influence of these parameters is analytically and numerically studied.

## 3. Mathematical Modeling

The aim of this section is to provide, at the complexity level required by the aim of this paper, analytic formulations for estimating the initial magnetization curve of ferromagnetic materials, the magnetic field generated in the vicinity of a permanent magnet, and the hysteresis losses that are produced by a ferromagnetic hysteresis rod.

### 3.1. Initial Magnetization Curve

In this subsection an analytic formulation is presented that describes the initial variation of the magnetic flux density inside an infinitely elongated hysteresis rod, *B*, as a function of the external applied field, *H*. To the authors' knowledge, a unique expression that can provide the variation of the magnetic flux density generated inside a material for the whole range of applied external magnetic field is not available. Nevertheless, to this aim, the whole magnetic range can be split into three parts, which can be separately modeled. The first region of the curve was first modeled by the Rayleigh relationship; the intermediate part can be approximated by the Polley, Becker and Doring general formulation; and the upper regions, where the magnetism inside the body is approaching saturation, can be well estimated by the equation used by Weiss [6, 12], as follows:

(1a) B=μiH+νH2, 0<H<H1,

(1b) =Bs(1−a0H−1−b0H−2−c0H−3−⋯)+κ0H,               H1<H<H2,

(1c) =Bs(1−a0′H−1−b0′H−2−c0′H−3−⋯),            H2<H<H3, 

where *H*_1_, *H*_2_, and *H*_3_ depend on each material and define the limits of each region; *μ*_*i*_ is the initial permeability of the material; *ν* the coefficient of irreversible changes in magnetic induction at the first portion of the curve; *B*_*s*_ the saturated magnetic flux density generated inside the material; *H* the external magnetic field that is applied to the body; and *a*_0_, *a*_0_′, *b*_0_, *b*_0_′, *c*_0_, *c*_0_′,… and *κ*_0_ are the coefficients of the corresponding expansions.

As it was explained before, in designing the PMASS, utilizing hysteresis rods, it is preferable to take advantage of the second part of the initial magnetization curve, (1b). Obviously, by increasing the number of terms in the corresponding expansion, the accuracy of the results is increased. However, it was observed that, for the purpose of this paper, the equation can provide a satisfactory representation of the effect by neglecting terms of higher order than *H* ^−1^, which is:

(2) $$\begin{array}{c} B=B_{s}\left(1-a_{0}H^{-1}\right)+\kappa_{0}H,\;H_{1}<H<H_{2}. \end{array}$$

Therefore, for estimating the initial magnetization curve of a material, using this equation, the two corresponding magnetic flux density constants (*a*_0_ and *κ*_0_) and saturated magnetic flux density, *B*_*s*_, of those materials must be known. These parameters for seven different materials were obtained from the corresponding experimental magnetization data of each material [5, 13–15], using least square method, and they are presented in Table 1. However, it has to be taken into account that not a unique value can be attributed to every single material as these characteristics are not just a function of the material but of the heat treatment and manufacturing processes as well.

Finally, the formulation was applied to the case of four existing satellites, whose in-flight data were published. If the margins that the simplifications in the physical and mathematical model are considered, a good agreement can be observed between the predicted damping time and the in-flight data.

**Figure 2:**
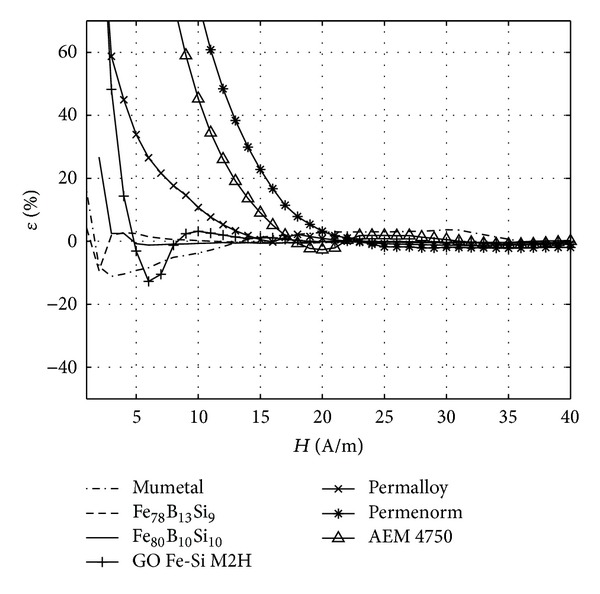
Variation with the applied magnetic field, *H*, of the relative error, *ε*, of the analytic model for the second part of the initial magnetization curve of seven different materials, as indicated in the legend.

For evaluating the accuracy of the proposed model, the results obtained from (2), *B*_an_, were compared with the experimental ones, *B*_exp⁡_, through the calculation of the relative error with regard to the saturated magnetic flux density generated in each material (*ε* = (*B*_exp⁡_ − *B*_an_)*B*_*s*_^−1^ × 100), as is presented in Figure 2. For all the materials the considered error tends to zero when the external magnetic field is increased. This is due to the initial part of the curve that is not accurately modeled by (2). Nevertheless, this problem has not been solved because, as explained before, this part of the curve is not within the interest of this work. However, it has to be mentioned that some materials like AEM-4750, Permenorm, and to some extent, GO Fe-Si have this part of their initial magnetization curve more extended, so the percentage of error can be high for a bigger range of external magnetic field for these materials.

As it is seen in Table 1 and Figure 2 the studied materials are mumetal, Fe_78_B_13_Si_9_, Fe_80_B_10_Si_10_, and GO Fe-Si, as they were proposed by [5], and Mo-Permalloy-79, Permenorm, and AEM-4750 are included because they were used on-board the satellites studied in this paper.

It is obvious that in satellite applications, the external magnetic field that the rods will experience is the geomagnetic field of the Earth at the corresponding position of the satellite. Based on the data provided in [18], the strength variation of the geomagnetic field at the distance *R* from the center of the Earth varies in the range [1,2] × ((7.71 × 10^15^)/(*μ*_0_ *R* ^3^)) A/m, where *μ*_0_ is the vacuum permeability. Hence, at 600 km altitude orbit the magnetic field of the Earth varies between 20 and 40 A/m.

Therefore, for obtaining an optimum design, the chosen rod should be magnetized up to its second level of magnetization when the applied field is within 20 to 40 A/m. However, the influence of the permanent magnet on the ferromagnetic materials should also be taken into account.

One has to bear in mind that since a permanent magnet applies a constant field over the rods, by fixing some parts of the domain it reduces the corresponding hysteresis loss of the rod. The most practical way to take the effects of the permanent magnets into account is to subtract their corresponding magnetic field from the oscillating field. However, it has been observed that in some existing satellites (e.g., Delfi) the generated magnetic field of the permanent magnet, *H*_pm_, was much bigger than the geomagnetic field. This case is presented separately in Section 4, where the proposed analytical model is applied to some selected existing satellites, including Delfi.

### 3.2. Magnetic Field Generated by a Permanent Magnet

In this subsection, a mathematical model to evaluate the magnetic field generated by the permanent magnet at its vicinity is presented. The magnetic field vector generated at a given point by a permanent magnet in its vicinity, **H**_pm_, can be expressed as the negative gradient of a potential function, which is called the scalar potential function of the permanent magnet, *ϕ*_pm_,

(3) $$\begin{array}{c} H_{\text{pm}}=-\nabla \phi_{\text{pm}}. \end{array}$$

This potential function at an arbitrary point P, located by the position vector **r** (with its origin at the center of the permanent magnet), can be evaluated through

(4) $$\begin{array}{c} \phi_{\text{pm}}=\frac{1}{4\pi }\int_{V_{\text{pm}}}\frac{r\cdot M}{r^{3}}\;\text{d}V^{\prime }, \end{array}$$

where **M** is the magnetization vector and *V*_pm_ the volume of the permanent magnet. Considering these relations, the magnetic field vector **H**_pm_ generated by a parallelepiped shape (3 cm × 1 cm × 1 cm) permanent magnet with a magnetization of 1 × 10^5^ A/m oriented along its longitudinal direction, inside the volume of a cubic satellite with 0.5 m side length, can be evaluated. According to this estimation the corresponding field of the permanent magnet inside the satellite varies from the maximum value of 5.2 × 10^4^ A/m, right above the permanent magnet to the minimum value of 0.054 A/m in the farthest point from the magnet inside the satellite.

### 3.3. Hysteresis Losses

In this section, a mathematical model for evaluating the variation of the hysteresis losses within a material as a function of the magnitude of the generated magnetic field inside a material, *H*_in_, and the maximum magnetic flux density, *B*_max⁡_, is defined for different portions of the initial magnetization curve of the material.

For the lower values of the applied magnetic field, where the initial magnetization curve obeys the parabolic relationship of Raleigh (1a), the values of hysteresis losses increase nearly proportional to *B*_max⁡_^3^, where *B*_max⁡_ is the maximum magnetic flux density generated inside the body. But for higher values of *H*_in_ the hysteresis losses are closer to *B*_max⁡_^2^. This variation in the middle point of a body, *W*_*h*__m_, for the first portion of the magnetization curve, was first evaluated by Raleigh, and for the second part by Steinmetz, which are presented here together,

(5a) $$\begin{array}{c} {W_{h}}_{\text{m}}=\frac{4}{3}\nu H_{\text{in}}^{3},\;0<H_{\text{in}}<H_{1}, \end{array}$$

(5b) $$\begin{array}{c} {W_{h}}_{\text{m}}=\eta B_{\max}^{m},\;H_{1}<H_{\text{in}}<H_{2}, \end{array}$$

where *η* is a proportionality constant and *m* is the corresponding power value, which varies depending on the material. The third part of the curve has not been modeled here as the external magnetic field that an Earth orbiting satellite might experience is not high enough to lead the magnetization of a ferromagnetic rod to enter this part of the magnetization curve. The values of *m* and *η* for the studied materials are presented in Table 1.

According to [6], the values of *m* are 1 ≤ *m* ≤ 2 for most of the materials. Therefore, in case that a more accurate value of this parameter for a particular material is not known, the two extremes of this range can be considered to evaluate a range for the hysteresis losses of the material.

Once the hysteresis losses in the middle point of the rod, *W*_*h*__m_, are determined, the hysteresis losses within the whole volume of the rod, *V*, can be evaluated by using

(6) $$\begin{array}{c} W_{h}=\kappa_{w}{W_{h}}_{\text{m}}V, \end{array}$$

where the coefficient *κ*_*w*_ ranges between 0.55 and 0.65 depending on the material [5]. So, a mean value of 0.6 is employed, which is expected to give acceptable results for all the materials. Therefore, according to (6) and (5a) and (5b), for evaluating the hysteresis losses, the magnitude of the magnetic field generated in a rod, *H*_in_, and the maximum magnetic flux density, *B*_max⁡_, should be determined.

These parameters, *H*_in_ and *B*_max⁡_, are both functions of the maximum external magnetic field, *H*_*a*_, and the shape and dimensions of the rod. This dependence is due to the demagnetization field generated inside the rod, which is explained in the following subsection.

### 3.4. Demagnetization Field

The magnetic flux density generated in a finite elongated rod is a function of its elongation as well. In this section, the influence of the elongation is evaluated through the assessment of the demagnetization field that is generated in a finite elongated body.

The demagnetization field is a magnetic field generated inside a body that opposes the applied magnetization and causes the net internal field to be less than the external applied magnetic field, which is written as

(7) $$\begin{array}{c} H_{\text{in}}=H_{a}-H_{d}, \end{array}$$

where *H*_in_ is the net magnetic field generated inside the finite elongated hysteresis rod, *H*_*a*_, the peak value of the applied oscillating magnetic field, and *H*_*d*_ is the demagnetization field generated inside the rod. In the case that the internal magnetic flux density is aligned with one of the main geometrical directions of the body, the corresponding homogeneous demagnetization field is proportional to the magnetic flux density generated inside the bar through the proportionality constant *N*_*d*_ and can be written as *H*_*d*_ = *N*_*d*_ *B*/*μ*_0_. Once the internal magnetic field generated in a body is known, the magnetic flux density of the body can be evaluated by using

(8) $$\begin{array}{c} B=\frac{\mu_{0}\left(H_{a}-H_{\text{in}}\right)}{N_{d}}. \end{array}$$

In bodies with a large elongation the problem of demagnetization is less significant, although the demagnetization field can never be ignored in measurements. Many analytic approaches have been carried out that provide reasonable approximation for estimating the demagnetization factor of different geometric shapes but apply only to diamagnets, paramagnets, and saturated ferromagnets [19–21]. However, they still can provide reasonable approximations of the demagnetization fields even for field values lower than the saturation. Based on experimental and analytic formulations, the demagnetization factor of two practical shapes—CCSR and TFR—of hysteresis rods is presented in Table 2.

Later, the maximum flux density that is induced inside a body can be obtained through the intersection of curve *B*(*H*) given by (2), which is the initial magnetization curve of a material, with curve *B*(*H*_in_) given by (8), which is the demagnetization field generated in a finite elongated rod. This is equivalent to solve the following system of equations:

(9a) $$\begin{array}{c} B_{\max}=B_{s}\left(1-a_{0}H_{\max}^{-1}\right)+\kappa_{0}H_{\max}, \end{array}$$

(9b) $$\begin{array}{c} B_{\max}=\left(H_{a}-H_{\max}\right)\mu_{0}N_{d}^{-1}, \end{array}$$

where the variable *B* in (2) and (8) is changed to *B*_max⁡_, as the intersection of the two curves corresponds to the maximum magnetic flux density generated inside the rod. Consequently *H* and *H*_in_, respectively, in (2) and (8), have been changed to *H*_max⁡_, which is the maximum internal magnetic field of a rod with demagnetization factor *N*_*d*_ under the maximum applied magnetic field *H*_*a*_ as follows:

(10) Hmax⁡=(−(Bs−μ0Nd−1Ha)  +(Bs−μ0Nd−1Ha)2+4(κ0+μ0Nd−1)a0Bs) ×(2(κ0+μ0Nd−1))−1.

Once *H*_max⁡_ is known, the corresponding induced magnetic flux density inside the rod can be evaluated through (9a). As it is an analytical relationship, it helps to reduce the calculation time and to study the influence of the parameters involved as well.

### 3.5. Estimating Damping Time

Once the maximum induced magnetic flux density in the rod, *B*_max⁡_, is known, the hysteresis losses can be evaluated through (5a) and (5b) and (6). Furthermore, by knowing the dissipating energy due to hysteresis losses as a function of the different characteristics of the material and its shape the design procedure can be conducted towards finding an optimum point. However, it has to be taken into account that, although the Steinmetz's law is valid for a variety of materials, its validity should be tested for new materials as the validity of the equation fails for materials with certain characteristic constricted loops [6].

If the dissipated energy in the hysteresis materials that are on-board the satellite is known, the rotational speed decay time of the satellite can be evaluated by analyzing the angular dynamic of the satellite. Assuming the satellite is rotating around one of its principal axes, the decreasing rate of the angular velocity of the satellite can be expressed according to the Euler angular equation of motion as

(11) $$\begin{array}{c} I\frac{\text{d}\omega }{\text{d}t}=-T, \end{array}$$

where **ω** is the angular velocity of the satellite, *I* the moment of inertia with respect to the rotation axis, and *T* is the component of the damping torque vector along the axis of rotation. The environmental torque can be neglected compared to the torque generated by the interaction of the ferromagnetic material with the geomagnetic field.

According to the in-flight data of some satellites that have utilized hysteresis rods for reducing their initial angular velocities, the angular velocity showed a linear variation with time, which means that the damping torque is a constant. Therefore, assuming that the magnetic field follows a complete hysteresis loop in each rotation of the satellite, the torque can be approximated as *W*_*h*_/2*π*. Thus, the time, *t*_*d*_, that takes the satellite to reduce its initial angular velocity, *ω*_0_, to the desirable angular velocity of *ω* is

(12) $$\begin{array}{c} t_{d}=\frac{2\pi I}{W_{h}}\left(\omega_{0}-\omega \right), \end{array}$$

where *W*_*h*_ is the resultant hysteresis losses due to the dissipation of energy inside all the ferromagnetic materials on-board the satellite.

## 4. Results

In this section, the following points are discussed: (A) the best on-board layout for the PMASS, (B) the initial magnetization curve of some materials as defined before, (C) the variation of the dissipated energy of different materials versus their body shape characteristics, and (D) estimation of the damping time for several existing satellites.

### 4.1. Influence of the Permanent Magnet

The intention of this subsection is to find the best on-board layout for the hysteresis rods with respect to the permanent magnet. Obviously, a layout arrangement through which the influence of the permanent magnet on hysteresis rods is as low as possible is the most desirable one. Thus, the influence of the permanent magnet on different types and positions of hysteresis rods should be studied.

It has been cleared so far that, because of the high values of demagnetization field that a TFR generates in the perpendicular direction to its plane, the magnetization vector in a TFR of soft ferromagnetic materials is constrained to lie in the longitudinal plane of the film. In fact, the demagnetization factor, *N*_*d*_, of a thin film perpendicular to its plane is unity, whilst if the magnetization lies in the film plane *N*_*d*_ is much smaller. And due to the fact that the magnetic field vectors generated by the permanent magnet are perpendicular to equatorial plane at this plane, so TFRs function more efficiently when placed in this plane. Nevertheless, this position is not the best for CCSRs, as the diameters of the commonly used CCSRs are at least one order of magnitude greater than the thickness of TFRs. This results in the creation of lower demagnetization fields in perpendicular direction to longitudinal axis in CCSRs rather than TFRs. Therefore, the best position for CCSRs is the farthest position from the permanent magnet.

**Figure 3:**
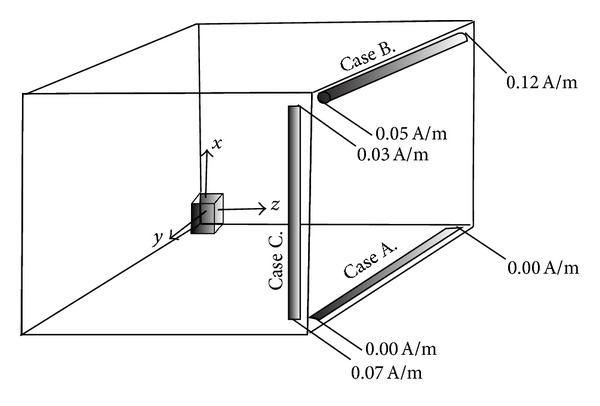
Layouts studied: Cases A–C, calculated value of the effective magnetic fields, *H*_pm_, generated by the permanent magnet at both ends of the rods.

Based on this knowledge, three different cases as a combination of position and shape of the rods (Figure 3) are studied in this section:

 Case A. A TFR in the equatorial plane of the permanent magnet, oriented along *y*-axis.
 Case B. A CCSR in the farthest position from the permanent magnet, oriented along *y*-axis.
 Case C. A TFR in the farthest position from the permanent magnet and oriented along the *x*-axis.

The position of the permanent magnets and hysteresis rods in all the studied cases, along with the corresponding values of the magnetic field generated by the permanent magnet at both ends of each rod, is shown in Figure 3. The permanent magnet for conducting these studies was considered to be the same magnet as the one explained in Section 3.2. The dipole direction of the permanent magnet is considered to be along the *x*-axis of the reference frame. The results of these cases are explained in the following paragraphs.

**Case A**

Using (3) and (4), the effective magnetic field of the permanent magnet that fixes some part of the domains along a hysteresis rod can be evaluated. However, it has to be taken into account that for evaluating the effective magnetic field that influences a thin film of hysteresis rod, the component of the field that is perpendicular to the plane of the film should not be considered in calculations.

Considering all these facts, the effective magnetic field of the permanent magnet that affects the domain distribution in a thin film, placed in this position, is zero.

**Case B**

In case of a CCSR, all the components of the field generated by permanent magnet should be considered effective; therefore, the best position for this rod would be the farthest position from the permanent magnet, as is shown in Figure 3. Placing the CCSR in this position, the effective magnetic field of a permanent magnet with the magnetization of 1 × 10^5^ A/m, evaluated by (3) and (4), showed that this field changes from *H* = 0.05 A/m in one end to 0.12 A/m in the other end of the rod.

**Case C**

Last case to be studied is a rod being oriented along the *x*-axis (parallel to the magnetization vector of the permanent magnet). Of course, for providing the best efficiency it has to be placed in the farthest position from the permanent magnet, as it is shown in Figure 3. Due to the high attributed demagnetization factor to TFRs in the perpendicular direction to their plane, the rod for this position was decided to be a TFR. As in this case some part of the magnetic field of the permanent magnet does not affect the rod, it is relatively more efficient for this position. However, the final decision depends on the size of both the rods and the magnet. So, for deciding the shape a separate analysis should be conducted for each shape. But it has not been done here to limit the length of the paper, and we have limited ourselves to the TFR case.

**Figure 4:**
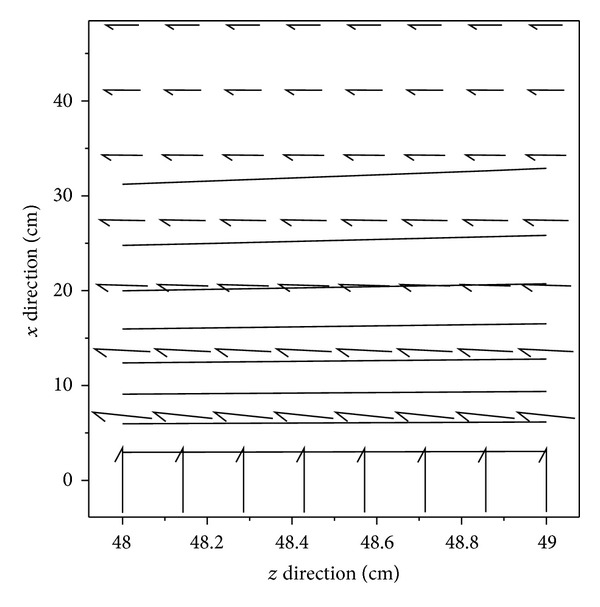
Constant magnetic scalar potential lines (solid lines) of the field generated by the permanent magnet along the plane of a hysteresis rod placed in the farthest position from the permanent magnet and oriented along the *x* direction ((Figure 3)-Case C). The direction of the arrows indicates the direction of the magnetic field of the permanent magnet over the plane of the rod, and their relative length indicates the relative magnitude of the magnetic field.

The effective magnetic field of the permanent magnet influencing this rod was calculated to be within [0.03,0.07] A/m. The variation of the corresponding magnetic field along with the magnetic flux constant lines is presented in Figure 4. As it can be seen in the figure, in some parts of the rod, the external field is parallel to the easy axis of magnetization of the TFR (longitudinal axis). Due to this reason and also to the higher value of the external magnetic field of permanent magnet at the vicinity of this rod, it is preferable to avoid placing any hysteresis rod along this axis, as the hysteresis rods along this axis result to be less efficient.

### 4.2. Approximating the Initial Magnetization Curve

The initial magnetization curve of seven materials (mumetal, Fe_78_B_13_Si_9_, Fe_80_B_10_Si_10_, Grain oriented Fe-Si, Mo-Permalloy-79, Permenorm, and AEM-4750) were approximated using (2). These materials were selected because of the following reasons: the first 4 materials were proposed by [5] due to their good performance; Permenorm and Mo-Permalloy-79 were studied because they were the most frequent materials used on-board reported existing satellites; and AEM-4750 because it is the material commonly used for hysteresis rods on-board small satellites by NASA. As it was explained in Section 3, for predicting the initial rotational speed decay time of the satellite, it is required to know the initial magnetization curve of the corresponding material. For the first estimation the data of Table 1 can be used. However, as the level of stress present inside the material, or the heat treatment procedure, can have a large influence on the shape of the initial magnetization curve of each material, a dispersion of values can be found for each of these characteristic coefficients. This topic is explained in [6].

**Figure 5:**
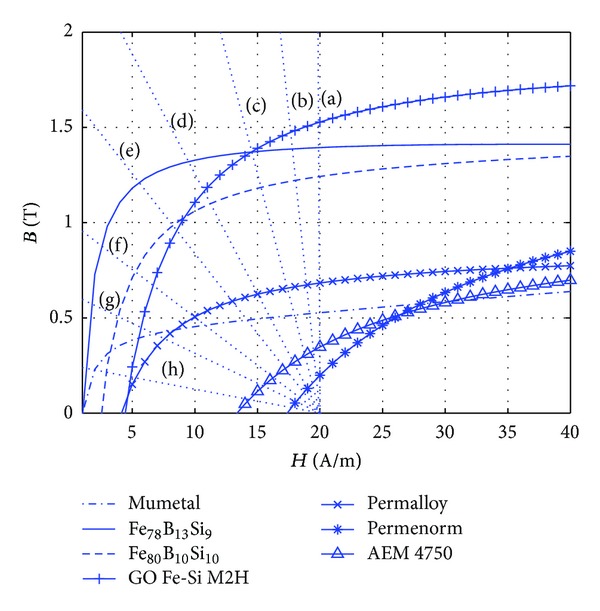
Variation of the magnetic flux density, *B*, as a function of the external magnetic field, *H*, along the initial magnetization curve, for seven different materials as is indicated in the legend. Dotted lines: demagnetization field generated inside the bodies with the demagnetization factor, *N*_*d*_, indicated by the label as (a) 1 × 10^−7^, (b) 2 × 10^−6^, (c) 5 × 10^−6^, (d) 1 × 10^−5^, (e) 1.5 × 10^−5^, (f) 2.5 × 10^−5^, (g) 4 × 10^−5^, (h) 1 × 10^−4^.

Using (2) and the data provided in Table 1, the initial magnetization curves have been computed and are presented in Figure 5. In order to evaluate the accuracy of the approximated curves based on (2), the experimental values of the corresponding materials have been compared with the approximated ones, and a good agreement was found just after the first part of the initial magnetization curve (*H* > *H*_1_), as in this part the concavity of the curve is upward and does not obey (2). However, due to its reversibility or the very little losses that happen in this part of the curve, this part is not of our interest. As explained in Section 3.1, the variation of the relative error, *ε*, with respect to the saturated magnetic flux density, *B*_*s*_, versus the applied magnetic field is presented in Figure 2.

The dashed lines in Figure 5 are the demagnetizing curves for several values of demagnetizing factors, *N*_*d*_. The intersections of these lines with the initial magnetization curves are the values of the maximum generated field, *H*_max⁡_, and maximum flux density, *B*_max⁡_, in the ferromagnetic hysteresis rods, as the solution of (9a) and (9b).

By approximating the initial magnetization curve, *B*(*H*), within the corresponding part of irreversible displacements of the domains (the second part of initial demagnetization curve), the maximum magnetic flux density, *B*_max⁡_, generated inside the ferromagnetic bodies for different values of demagnetization values is evaluated. However, it has to be taken into account that the approximations considered in Figure 5 are only valid within the irreversible region, as can be realized from Figure 2. Therefore, from these two figures it is clear that for producing more effective energy dissipation, effect the elongation of the body cannot be decreased below a certain limit for which the maximum magnetic flux density inside the material would be within the reversible part. This matter is explained further in the following paragraph.

The limit of the reversible part, *H* = *H*_1_, can be estimated from Figure 2 for a given error *ε* (e.g., *ε* < 10%). If these values are translated to Figure 5, then a limit region appears where the approximations of the initial magnetization curves are no longer valid. This limit almost coincides with the demagnetization curve for *N*_*d*_ = 1 × 10^−4^ when the external magnetic field amplitude is *H* = 20 A/m. This means that for staying within the second portion of the initial magnetization curve, which produces the maximum dissipated energy, the demagnetization factor of the body should not exceed 1 × 10^−4^. For each shape the value of demagnetization factor corresponds to some ratios of the specific dimensions of the body, which can be deduced from the expressions given in Table 2.

### 4.3. Dissipated Energy versus Body Shape Characteristics

In this section, the variation of the dissipated energy as a function of the thickness and diameter of TFR and CCSRs is studied. The performances of both shapes are compared and the length of the rod for each shape that gives the same dissipation is determined. It is shown that above this length the CCSRs demonstrate a more efficient behavior than the TFRs.

**Figure 6:**
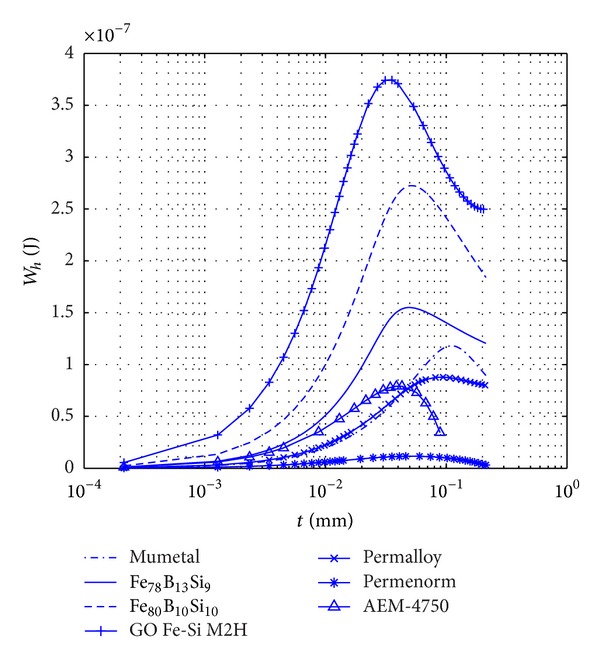
Variation with the thickness, *t*, of the dissipated energy per cycle, *W*_*h*_, in soft ferromagnetic TFRs, of 5 mm width and 0.2 m length, using Steinmetz power law, (5a) and (5b). External magnetic field peak value *H*_*a*_ = 20 A/m.

**Figure 7:**
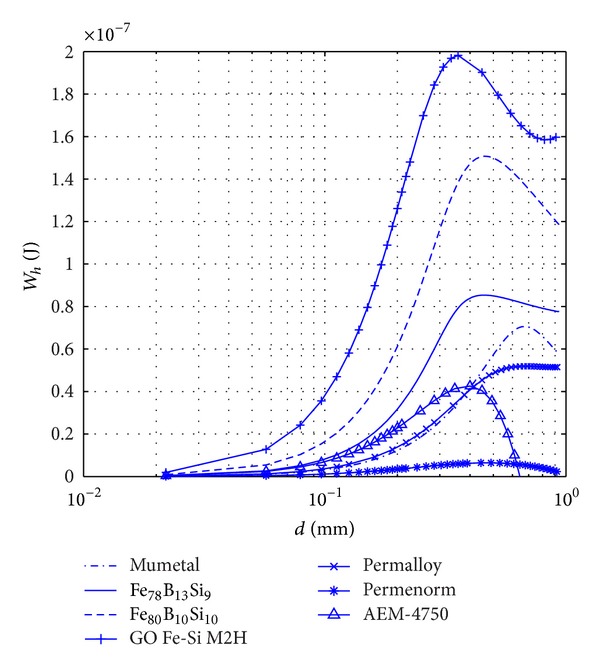
Variation with diameter, *d*, of the dissipated energy, *W*_*h*_, of CCSR. The same conditions as in Figure 6.

Once the maximum flux density, *B*_max⁡_, generated inside the body is known as a function of its thickness, *t*, or diameter, *d*, the variation of dissipated energy per cycle as a function of its thickness or diameter can be evaluated by using the power law of Steinmetz, (5b). Based on this relationship, the variation of the dissipated energy per cycle, *W*_*h*_, for different materials in the form of TFR and CCSRs as a function of their thickness and diameter are, respectively, plotted in Figures 6 and 7. For both cases, the length of the body was considered to be 0.2 m, and it was assumed that the internal magnetic field generated inside the body was within the range [*H*_1_, *H*_2_], while the peak value of the external magnetic field was *H*_*a*_ = 20 (A*⁄*m). As is shown in these figures, the dissipated energy attains a maximum value at a given thickness, *t*_max⁡_, or diameter, *d*_max⁡_, of the TFR or CCSR, respectively, as it was expected. *t*_max⁡_ or *d*_max⁡_ is hereafter referred to as the optimum thickness or diameter, respectively.

As it was explained in Section 4.2 through Figure 5, to keep the internal magnetic field generated in the body, *H*_in_, within the range of [*H*_1_, *H*_2_], the thickness has an upper limit. Exceeding this thickness *H*_in_ goes outside the range of [*H*_1_, *H*_2_], inside which the efficiency in dissipating energy is large. Outside this range the amount of dissipated energy would be much less and should not be considered in an optimum design.

**Figure 8:**
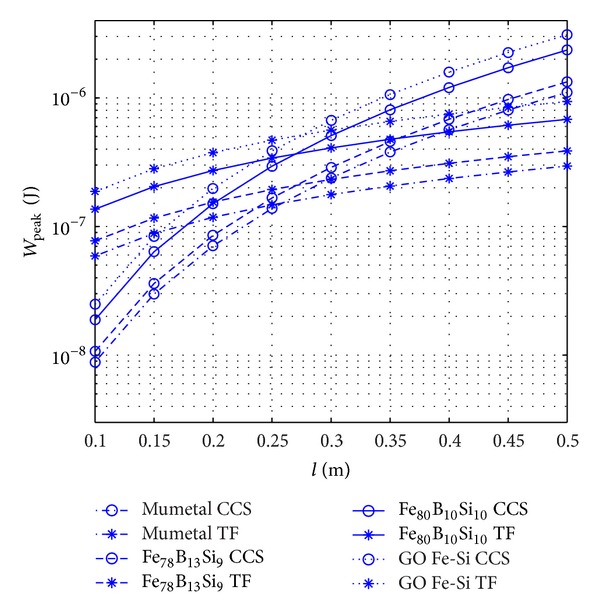
Variation with length, *l*, of the peak value of the dissipated energy, *W*_peak_, inside bodies of different materials and different cross sections, exposed to an oscillating magnetic field with a peak value *H*_*a*_ = 20 A/m. The width of the TFRs is *w* = 5 mm.

The maximum of dissipated energy that was attained in Figures 6 and 7 can be helpful to optimize the design. It would also be interesting to study the effect of the shape to find the most effective range of each shape. Comparing the respective dissipated energies in Figure 6 with the ones of Figure 7, it can be seen that the dissipated energy is larger in the case of TFRs. The maximum value of dissipated energy, *W*_peak_, was evaluated through Figures 6 and 7 for a given length, *l*, of the rod and width of TFR, *w*, while exposing to a specific applied field, *H*_*a*_, as explained in the preceding paragraphs. In Figure 8 the variation of the peak value of dissipated energy, *W*_peak_, is plotted against the length of the rod for four different materials and two different shapes of rod while the width of the thin films was kept equal to 5 mm. As shown in this figure, CCSRs present lower values of peak dissipated energy, *W*_peak_, when the length of the body is below some 0.26 m. The different influence of the length in both cases is due to the different dependence of the demagnetization factor on the length for the two cross-section types, as shown in the corresponding equations presented in Table 2.

Therefore, there is a point in Figure 8 where the two curves cross each other, hereafter denoted as the crossing point length. This length is expected to increase with the width, *w*, because in TFRs the peak value of dissipated energy increases with the width.

According to (7), another important factor in sizing the different parameters of hysteresis rods is the peak value of the applied oscillating field, *H*_*a*_. Obviously, the variation of this parameter is not within the control of the designer and depends on the altitude and rotational or oscillating behavior of the satellite. Therefore, by knowing the relevance of this parameter in the dissipating performance of hysteresis rods, the design should be tried to be as optimum as possible for the whole range of possible values of applied fields, *H*_*a*_, that the satellite will experience.

**Figure 9:**
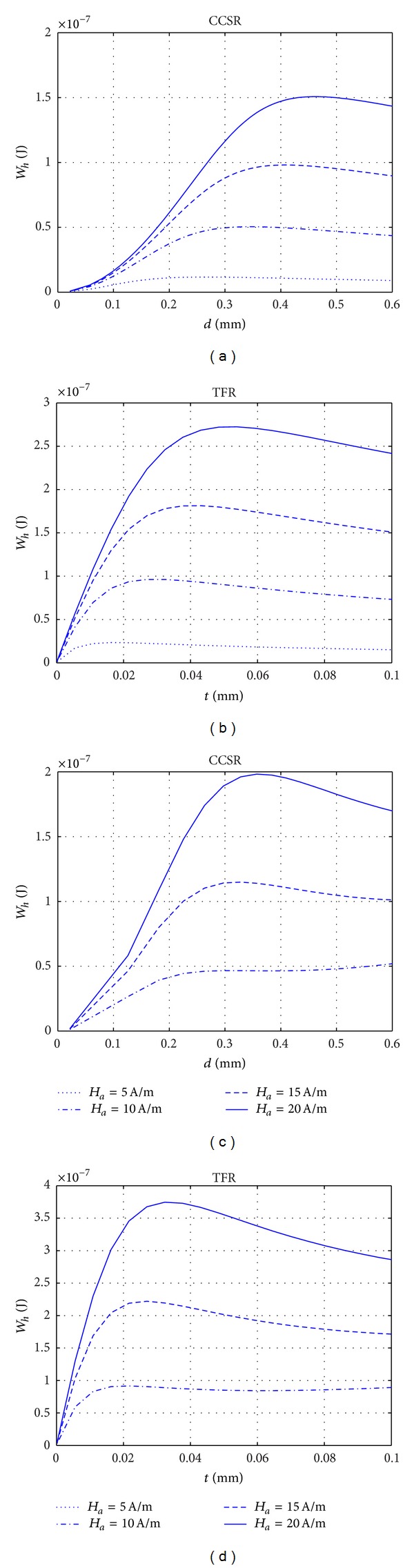
Variation of the dissipated energy, *W*_*h*_, as a function of thickness, *t*, or diameter, *d*, of TFR and CCSRs for materials Fe_80_B_10_Si_10_ and GO Fe-Si with length of 0.2 m, for different values of the peak applied field, *H*_*a*_. (a) CCSR, Fe_80_B_10_Si_10_, (b) TFR, GO Fe-Si, (c) CCSR, GO Fe-Si, and (d) TFR, GO Fe-Si.

To show the influence of the applied field, *H*_*a*_, in Figures 9(a)–9(d) the variation of the dissipated energy, *W*_*h*_, is plotted as a function of the diameter, *d*, or thickness, *t*, of CCSRs or TFRs for different peak values of the applied oscillating field, *H*_*a*_, and for two different materials (Fe_80_B_10_Si_10_ and GO Fe-Si). These data were obtained for TFRs with 0.2 m length and 5 mm width. In Figures 9(c) and 9(d), external magnetic field values in the range *H*_*a*_ < 10 A/m have not been considered, as for this material in this low range of applied field the dissipation cannot be calculated by using (5b) because it is not valid in this range. Furthermore, this range is not relevant, as a clear maximum does not appear. The results in this figure show that both the peak values of dissipated energy, *W*_peak_, and its corresponding optimum thickness or diameter depend on the strength of the applied oscillating fields.

**Figure 10:**
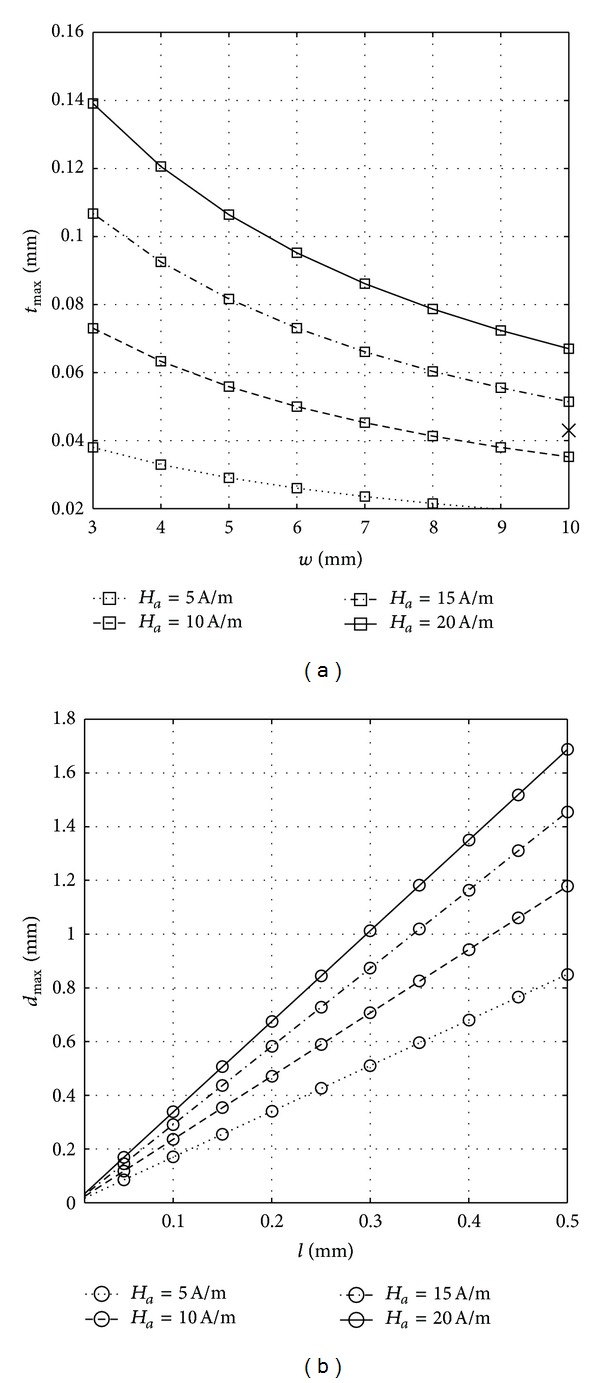
(a) Variation of the optimum thickness, *t*_max⁡_, of TFR, 0.2 m long, with the width, *w*, for peak values of applied field *H*_*a*_ = 5, 10, 15 and 20 A/m. (b) Variation of the optimum diameter, *d*_max⁡_, of CCSRs with the length of the body, *l*, in the same conditions as (a). Material: mu-metal.

Knowing the fact that the optimum diameter and thickness of CCSRs and TFRs depend on the magnitude of the magnetization field, thus it is desirable to conduct the design to a point where the influence of *H*_*a*_, which is an uncontrollable parameter, is lower. For doing so, the variation of the optimum thickness and diameter of TFR and CCSR, respectively, as a function of the width of the film and the length of the cylinder, for four different values of *H*_*a*_ was studied. The results are shown in Figure 10. As the behavior of all the materials concerning these parameters is the same, Figure 10 is plotted for just one material (mumetal).

As shown in Figure 10(a), by increasing the width of the TFR both the value of the optimum thickness, *t*_max⁡_, and the influence of the applied field, *H*_*a*_, on it decrease. This can be considered as an advantage for TFRs, because on one hand, a higher value of width leads to a higher value of dissipated energy in the rod, according to (6). On the other hand, the optimum values of thickness for different values of applied fields for higher values of width are concentrated in a smaller range which leads the selected design value to be closer to the optimum value of the in-orbit applied fields.

The variation of the optimum diameter as a function of the length of CCSRs for four different values of the applied field is shown in Figure 10(b). As is seen in this figure, on one hand by increasing the length of the CCSR the values of *d*_max⁡_ increase proportionally. On the other hand, by increasing the length the values of *d*_max⁡_ for different values of the applied field diverge, as the slope increases when the applied magnetic field, *H*_*a*_, increase. This in fact means that for the higher values of length the influence of *H*_*a*_, which is an uncontrollable parameter, in determining the optimum diameter increases. This behavior can be considered a disadvantage for CCSR as the increase of length that was previously seen to result in the increment of the dissipated energy (Figure 8) is accompanied with an adverse effect in the convergence of the optimum diameter for different values of the applied magnetic field.

Therefore, for TFRs, the thickness can almost always be designed very close to the optimum values due to the convergence of the optimum thicknesses for different applied fields by increasing the width of the body. However, for CCSR by increasing the length, the optimum diameters under the influence of different applied fields diverge. One has to bear in mind that higher values of width and length are more desirable, as they increase the volume and consequently dissipated energy. It has also to be kept in mind that the CCSRs are specially effective for higher lengths of rod compared to TFRs (Figure 8).

**Figure 11:**
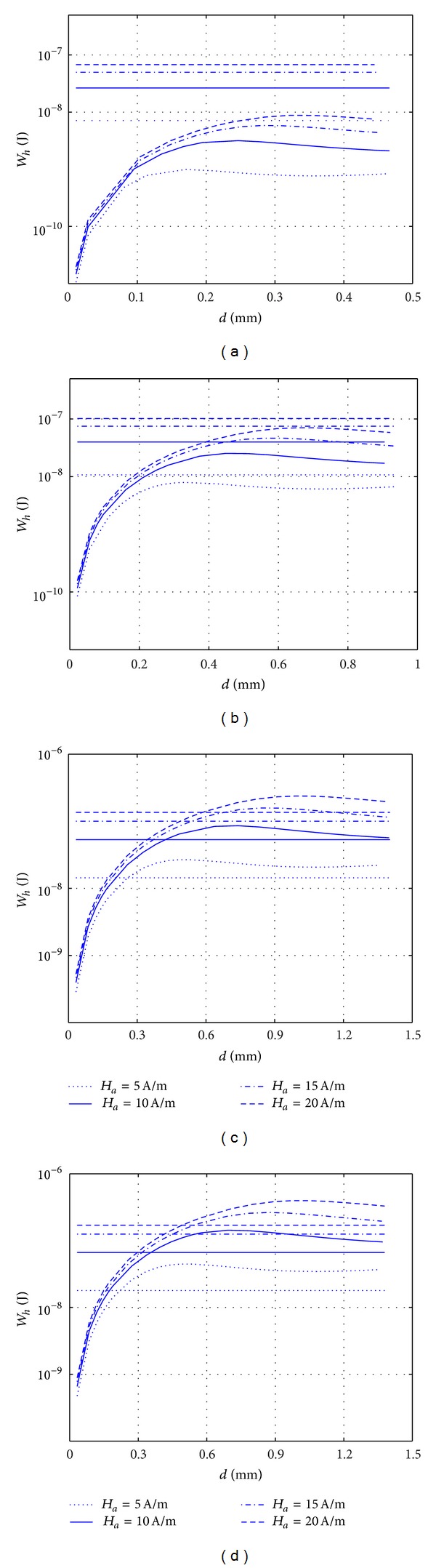
Variation with the diameter, *d*, of the dissipated energy, *W*_*h*_, within a CCSR, for several values of their length, *l*, (a) 0.1 m, (b) 0.2 m, (c) 0.3 m, and (d) 0.4 m, for four different peak values of applied field, *H*_*a*_ = 5, 10, 15, and 20 A/m. Horizontal lines: energy dissipated inside a TFR with width *w* = 10 mm and thickness *t* = 0.043 mm.

Thus, for CCSRs it is not always possible to design the diameter close to the in-orbit optimum value. This leads to the ambiguity whether the CCSRs still remain more efficient for higher lengths of the body compared to TFRs. As the diameter cannot be chosen equal to the optimum diameter and depending on the peak value of the real applied field that it can experience in orbit, the designed diameter might be off from the corresponding optimum diameter for that field. So, in order to make sure that the CCSRs are more efficient for the higher lengths of the body, the dissipated energy generated by different diameters of cylindrical bars should be compared with the dissipated energy generated by a TFR with the optimum dimensions that is obtained from Figure 10(a), for different values of the external applied magnetic field. In Figure 10(a), the cross sign is indicating the optimum value. The result of this comparison is shown in Figures 11(a)–11(d).

The variation of the dissipated energy of CCSR as a function of its diameter, for four different peak values of applied field, is plotted in Figure 11 along with the dissipated energy within a TFR with optimum dimensions obtained from Figure 10(a). Four different values of *l* = 0.1, 0.2, 0.3, and 0.4 m for rod length are considered, respectively, in Figures 11(a), 11(b), 11(c), and 11(d). The dimensions of the TFRs considered are *w* = 10 mm and *t* = 0.043 mm which are the optimum width and thickness obtained from Figure 10(a). The amount of energy dissipated by CCSR and TFRs for different values of the applied field and rod length can be compared by using Figures 11(a)–11(d) to choose the more efficient shape and dimension for the hysteresis rods.

As shown in Figure 11(a), the amount of energy dissipated in the CCSR is far below the one in TFRs, all over the range of its considered diameters for all the values of applied fields. As the length is increased (Figures 11(b) and 11(c)), the difference is reduced, and in some cases the behavior is reversed over a portion of diameter range. Increasing the length of the rod to 0.5 m the amount of energy dissipated in CCSR is the largest in almost all the range of the considered diameter of the body. Through this result the ambiguity that appeared previously can get clear by confirming that for the higher lengths of body (above the crossing point length), CCSRs display a better performance than TFRs, even when the diameter is not selected equal to *d*_max⁡_.

### 4.4. Estimated Damping Time

The aim of this section is to apply the energy dissipation model, given by (1a), (1b), and (1c) to (12), to estimate the effect on a satellite rotational speed decay time. As it is mentioned in Section 3.5, the time for a satellite to reduce its initial angular velocity to a desired one can be estimated through (12). Using this equation, the damping time for four satellites that have already flown was evaluated analytically and compared with their corresponding in-flight data. In this way the validation of the proposed model can be tried, and the efficiency of the above mentioned satellite PMASS designs can also be studied.

The in-flight data of existing selected satellites that have been used to perform this study are presented in Table 3, including their corresponding hysteresis material; rod shape; their cross-section areas, *A*; length, *l*; number of rods, *n*_*r*_; damping time, *t*_*d*_, needed to decrease its angular momentum a given amount *I*Δ(*ω*); the corresponding orbital radius, *R*; the dipole moment of the permanent magnet, *m*_pm_; and the on-board arrangement of the bars with respect to the permanent magnet.

It was observed that in some of the four satellites considered, the elongation of the rods was in a range of values such that the maximum internal magnetic flux density, *B*_max⁡_, of the rods was in the first part of the initial magnetization curve (thus working in the almost reversible range, suggesting a poor efficiency). To assess the behavior of the satellite in this range, it was required to evaluate the variation of the magnetization curve within this region, which has been modeled by (1a). To do this, the values of *μ*_*i*_ and *ν* for the materials that were implemented on these four satellites were estimated based on experimental data, [4, 13–15, 22, 23] that are presented in Table 4.

In the following paragraphs, an analysis of the selected existing satellites is presented.

*TRANSIT.* The TRANSIT satellite series were the first satellite navigation systems that were developed by the Applied Physics Laboratory (APL) of the Johns Hopkins University for the US Navy [22]. The satellites were equipped with 8 CCSRs, made up of magnetic material AEM-4750. As the orbital radii of these satellites were about *R* = 804 km, the average of the magnetization field in the orbit was evaluated to be about 25 A/m. As shown in Figure 5, the material AEM-4750 under the influence of an external magnetic field of *H* = 25 A/m is still far below its saturation (*B* = 0.5 T ≪ *B*_*s*_ = 1.04 T). Therefore, the use of demagnetization factors given by Table 2 in the corresponding equations would be a significant source of error; thus a modification of this model was needed. After some trials, it was found that the most simple change that gives satisfactory results is to consider a coefficient, *α*, as follows:

(13) $$\begin{array}{c} \alpha =0.73\;\text{atan}\left(4.91\frac{B}{B_{s}}\right). \end{array}$$

This modification of the demagnetization factor for a CCSR is given by

(14) $$\begin{array}{c} N_{d}=\alpha \frac{4.02\log_{10}^{e}-0.185}{2e^{2}}. \end{array}$$

By applying this demagnetization factor, the magnetization, *H*_in_, evaluated inside an AEM-4750 rod with elongation *e* = 248 is more in accordance with the experimental values presented in [4].

**Figure 12:**
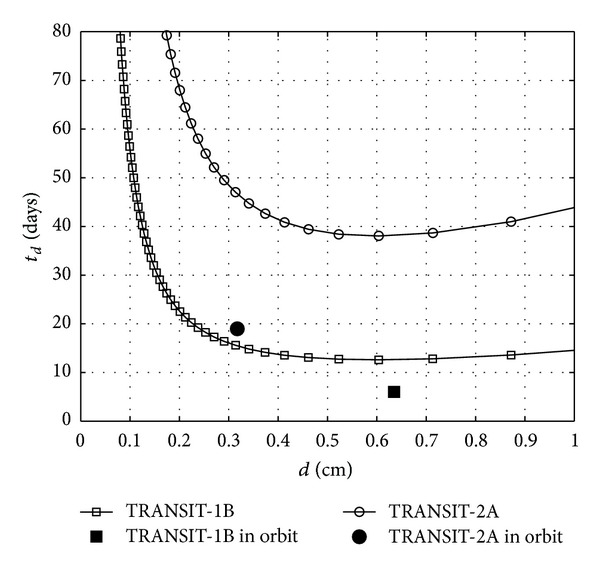
Variation of the damping time, *t*_*d*_, with the diameter of the hysteresis rods, *d*. Square: in-orbit data of TRANSIT-1B, circle: TRANSIT-2A.

By using the demagnetization factor and data of TRANSIT-1B and TRANSIT-2A in (12), the detumbling time, *t*_*d*_, as a function of the diameter of the rods is calculated and the results are shown in Figure 12. It can be appreciated that the estimation is in a good agreement with the in-flight data. Although the estimated damping time is bigger than that during the flight, which in case of TRANSIT-2A is more critical. This is because the damping effects of eddy current and shorted coils, which were wounded around a part of the hysteresis rods in case of TRANSIT-2A, were not considered in the model. These effects should amplify the damping effect of the permeable cores and are not included in the analytic model. Due to this, it was expected that the estimated values be larger than the real ones. It has to be mentioned that in the calculation of hysteresis losses through the rods, the value of *κ*_*w*_ in (6) was considered to be 0.73, according to the data published in [22].

As shown in Figure 12, the sizing of the rods seems to be close to the optimum and was improved in the case of TRANSIT-2A, although, according to the calculations in this paper, the optimum diameter is somewhere between the assigned values of the two satellites.

*Delfi.* Delfi-C3 was a nanosatellite developed by the Faculties of Aerospace Engineering and Electrical Engineering of Delft University [24]. The elements of the PMASS of this satellite were a permanent magnet and two CCSRs.

As it was also explained in Section 3, a nonvarying external magnetic field can cause a part of the domains to get fixed with respect to each other, so those domains do not participate in the dissipation action anymore. In the case of Delfi, due to the presence of a relatively large permanent magnet, almost all the domains in the rods should have remained fixed. This means that the domains are no longer moving in the two first parts of the hysteresis curve presented in Figure 1. Therefore, the contribution of the hysteresis rods in despinning the satellite is due to the movement of the domains that happen in the third part of the hysteresis curve.

As not any practical formulation for evaluating the hysteresis loss in this third region is available in the literature, to the authors' knowledge, the same equation that was used for estimating the losses in the first region of the curve, (1a), was also used. In both regions the amount of energy losses is much less than in the second region, as shown in Figure 1.

In order to estimate the energy losses in this region using (1a), first the total external magnetic field that the hysteresis rods are exposed to should be evaluated. In the case of Delfi, the hysteresis rods were exposed to the magnetic field of the permanent magnet plus the geomagnetic field which is not constant in magnitude and orientation along the orbit. The constant magnetic field due to the permanent magnet is estimated to be about 60 to 150 A/m, respectively, for each rod. The difference is due to their relative position with regard to the permanent magnet. Later, due to the motion along an orbit of 635 km altitude, the rods got exposed to a nonconstant external field of the order of 27 A/m. Finally, considering the peak value of *H*_*a*_ = 87 and 177 A/m for the applied magnetic field for each rod, the variation of the magnetic induction inside the rods for different values of the rod elongation was evaluated.

The part of induction that contributes to the dissipation is obtained by subtracting the induction that was due to the permanent magnet from the ones evaluated as a result of total external magnetic field for different values of demagnetization factor. The induction that was due to the permanent magnet was evaluated separately and resulted to be about 1.07 and 1.27 T for each bar.

**Figure 13:**
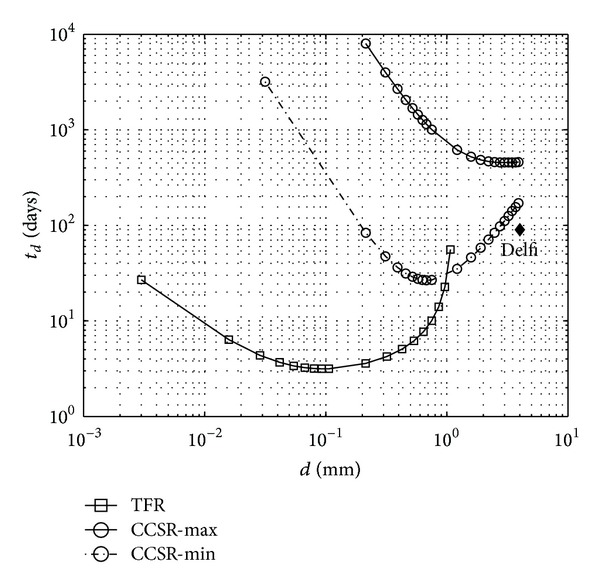
Variation of the damping time, *t*_*d*_, with the diameter, *d*, of the CCSR (Delfi-C3 design), and thickness, *t*, for TFRs (proposed option), for Delfi-C3 satellite. Rhombi: in-orbit data. Max and min correspond to *μ*_*i*_ = 3.8 × 10^−3^ Tm/A, and *μ*_*i*_ = 12.6 × 10^−3^ Tm/A, respectively.

The energy losses due to the varying magnetic induction inside these rods, *W*_*h*__m_, can be estimated using (5a), and the corresponding magnetic field, *H*, that results in such inductions should be evaluated using (1a), by applying the values of *ν* and *μ*_*i*_ provided in Table 4. The required damping time for Delfi, *t*_*d*_, is calculated for both TFR and CCSRs as a function of the thickness of the bodies and the results are shown in Figure 13 along with in-flight data of this satellite. As shown in Figure 13, and as explained in Section 4.1, if the cylindrical rods are placed in a plane perpendicular to the permanent magnet, this design would not be a very efficient one as, depending on the magnetic dipole of the permanent magnet, the CCSRs get saturated to a certain point. Nevertheless, the TFRs for such a configuration would be much more efficient, as shown in Figure 13. It has to be mentioned that the estimated damping times with the CCSRs are very rough. This is because a model for evaluating the loss in the third part of the initial magnetization curve was not found. Therefore, the assumptions that were explained in the preceding paragraphs were introduced. However, the intention of this paper is not to study the first and last regions of the hysteresis graph, so the results of this rough estimation were considered acceptable enough for our purpose.

The minimum and maximum variation curves of cylindrical case correspond to the extreme values of the initial relative permeability region of Permenorm, which was considered to be in the range [3000, 10000], as is also presented in Table 4. Later, the corresponding curves of the TFR cases were obtained through (5b) for *H*_1_ < *H* < *H*_2_.

As shown in Figure 13 for CCSR and same material, a clear reduction of the damping time can be obtained, by reducing the rod diameter to a few parts of a millimeter. Also employing the same number of the rods, but using TFRs, the minimum damping time decreases from about 27 days to some 3 days.

**Figure 14:**
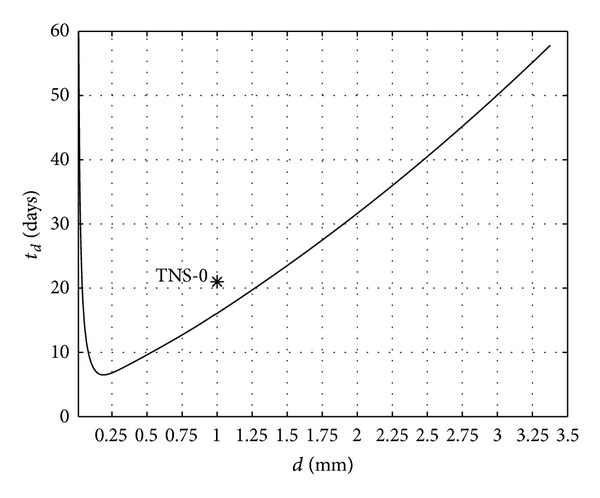
The same as Figure 12 for the satellite TNS-0. The in-flight data is indicated by the star.

*TNS-0.* TNS-0 was the first Russian nanosatellite [25] that implemented a PMASS. It carried 8 hysteresis rods with rectangular cross-section and a permanent magnet. The corresponding information of the satellite is shown in Table 3. The variation of the estimated damping time with the thickness of the rods predicted by the model for this satellite, along with in-flight data, is presented in Figure 14. The agreement between the results of the model and the in-flight data is not bad. It can be also noted that the design has some margin for improvement: by reducing the thickness of the films from 1 mm to about 0.2 mm, the estimated damping time can be reduced from 14 to 8 days.

## 5. Conclusion

In this paper, the variation of the damping energy capacity of different materials as a function of their elongation for rods of different cross-section has been modeled and evaluated analytically. Based on this formulation, an optimum design concerning the layout and shape of the hysteresis rods for satellites with different dimensions has been proposed.

By changing the thickness and diameter of, respectively, thin film and circular cross-section rods, a maximum dissipating energy was obtained. It was observed that in addition to the physical properties of the rod, the maximum value of the applied field also influences the maximum value of dissipating energy and its corresponding thickness or diameter of the rods.

Furthermore, comparing the dissipated energy resulting from thin films with cylindrical rods, a length was obtained, below which the thin film rods represent a more efficient behavior.

Also, it was shown that by a suitable arrangement of the on-board magnetic material, the efficiency of the PMASS can be improved significantly.

## Figures and Tables

**Table 1:** Characteristic coefficients of materials.

Material	*B*_*s*_ (T)	*a*_0_ (A/m)	κ_0_ × 10^3^ (T · m/A)	η (J/T^*m*^)	*m*
Mumetal	0.45	1.02	5.0	12.0	1.97
Fe_78_B_13_Si_9_	1.49	1.02	−1.0	5.6	1.29
Fe_80_B_10_Si_10_	1.39	2.46	1.0	13.0	1.43
GO Fe-Si	1.99	4.37	−1.4	20.0	1.72
Mo-Permalloy-79	0.86	4.12	0.1	7.0	1.60
Permenorm	1.53	17.27	−0.5	13.0	1.35
AEM-4750	1.04	13.37	0.1	35.0	2

**Table 2:** Demagnetization factor, *N*_*d*_, for different rod cross section shapes. *e* is the elongation, *w* the width of TFR, *t* the thickness, all expressed in (m).

Shape	*N*_*d*_	Reference
CCSR	$\frac{4.02\;\;\log_{10}^{e}-0.185}{2e^{2}}$	[16]
TFR	(0.057 *w* × 10^3^ + 0.2) *t*	[5]

**Table 3:** In-flight data of existing selected satellites.

Satellite	Material	Shape	*A*	*l*	*n*_*r*_	*I*Δω	*t*_*d*_	*R*	*m*_pm_	On-board arrangement
			(mm^2^)	(m)		(kgm^2^/s)	(days)	(km)	(Am^2^)	
TRANSIT-1B	AEM-4750	CCSR	32	0.78	8	16.86	6	804	N.A.	N.A.
TRANSIT-2A	AEM-4750	CCSR	8	0.78	8	50.88	19	804	N.A.	N.A.
DELFI-3C	Permenorm	CCSR	11	0.07	2	0.0027	86	635	0.3	In a plane perpendicular to the permanent magnet axis and passing through its center
TNS-0	Mo-Permalloy	TFR	2 × 1	0.12	8	0.076	21	350	2.2	In a plane perpendicular to the permanent magnet

**Table 4:** Properties of magnetic materials used in studied satellites.

Material	μ_*i*_ (Tm/A)	ν (T(m/A)^2^)
AEM-4750	8.5 × 10^−3^	3.95 × 10^−4^
Mo-Permalloy	2.5 × 10^−2^	1.1 × 10^−2^
Permenorm	(3.8,12.6) × 10^−3^	(4.1 × 10^−4^, 1.55)

## Nomenclature

*a*_0_, *a*_0_′: Magnetic flux density proportionality constant for different materials [A/m]
*B*: Magnetic flux density inside a material [T]
*B*_Earth_: Potential magnetic flux density of the Earth [T]
*B*_max⁡_: Maximum magnetic flux density generated inside a hysteresis rod [T]
*B*_*s*_: Saturated magnetic flux density of a material [T]
*b*_0_, *b*_0_′: Magnetic flux density proportionality constant for different materials [(A/m)^2^]
*c*_0_, *c*_0_′: Magnetic flux density proportionality constant for different materials [(A/m)^3^]
*d*: Diameter of the circular cross-section hysteresis rod [m]
*d*_max⁡_: Optimum diameter of circular cross-section rod [m]
*e*: Elongation or aspect ratio of the hysteresis rod
*H*: External magnetic field strength [A/m]
*H*_*a*_: Maximum value of the applied magnetic field [A/m]
*H*_*d*_: Demagnetizing magnetic field inside a hysteresis rod [A/m]
*H*_in_: Generated magnetic field inside a hysteresis rod [A/m]
*H*_max⁡_: Maximum generated magnetic field inside a hysteresis rod [A/m]
**H**_pm_: Magnetic field vector generated by a permanent magnet [A/m]
*I*: Satellite moment of inertia with respect to the axis of rotation [kg · m^2^]
*l*: Length of the hysteresis rod [m]
**M**: Magnetization vector of a permanent magnet [A/m]
*m*: Exponent value of maximum magnetic flux density in Steinmetz law
*m*_pm_: Magnetic dipole of the permanent magnet [Am^2^]
*N*_*d*_: Demagnetization factor of a hysteresis rod
*R*: Orbital radius of the satellite [m]
**r**: Position vector [m]
*T*: Component of the damping torque vector along the axis of rotation [Nm]
*t*: Thickness of a thin film hysteresis rod [m]
*t*_*d*_: Damping time [s]
*t*_max⁡_: Optimum thickness of the thin film hysteresis rod [m]
*V*: Volume of the hysteresis rod [m^3^]
*V*_pm_: Volume of the permanent magnet [m^3^]
*W*_*h*_: Hysteresis loss energy of a hysteresis rod [J]
*W*_*h*__m_: Hysteresis loss energy of a hysteresis rod at the middle point of the rod [J/m^3^]
*W*_peak_: Peak value of dissipated energy [J]
*w*: Width of the thin film of hysteresis rod [m]
*α*: Correction coefficient for the demagnetization factor
*μ*_0_: Permeability of the vacuum 4*π* × 10^−7^ [Tm/A]
*μ*_*i*_: Initial permeability of a ferromagnetic material [Tm/A]
*ν*: Irreversible changes coefficient in the lower portion of magnetization of materials [Tm^2^/A^2^]
*η*: Proportionality constant in Steinmetz law [J/T^*m*^]
*κ*_0_: Magnetic flux density proportionality constant for different materials [Tm/A]
*κ*_*w*_: Coefficient of the dissipation of energy through the whole volume of the hysteresis rod
*ω*: Satellite's angular velocity [rad/s]
*ω*_0_: Satellite's initial angular velocity [rad/s]
*ϕ*_pm_: Permanent magnet scalar potential function [A].

### Acronyms

PMASS: Passive Magnetic Attitude Stabilization System
TFR: Thin Film Rod
CCSR: Circular Cross-Section Rod.
